# Impact of physical exercise on sleep quality in college students: A Chain mediating role of self-efficacy and emotional control

**DOI:** 10.1371/journal.pone.0340208

**Published:** 2026-01-29

**Authors:** Wen-hao Zhang, Wei-dong Zhu, Hu Lou, Ding-you Zhang, Fan-zheng Mu, Xin-yu Zhang, Yu-han Li, Hao-jie Zuo, Qi Liu, Mo-han He, Jia-qiang Wang, Chen-xi Li, Hao-yu Li, Ning Zhou, Yao Zhang, Wei Wang, Xiao-yu Wang, Lan-lan Yang, Bo-chun Lu, Lin-lin Zhao, Shan-shan Han, Ya-xing Li, Yang-sheng Zhang, Ling-li Xu, Yu-yan Qian, Chuan-yi Xu, Han Li, Shuo Feng, Qing Zhang, Jian-gang Sun, Adenan Ayob, Bo Li, Lei Ding

**Affiliations:** 1 Institute of Sports Science, Nantong University, Nantong, China; 2 School of Physical Education, Shanghai Normal University, Shanghai, China; 3 Physical Education University, Shangqiu University, Shangqiu, China; 4 School of Physical Education, Nanjing Xiaozhuang University, Nanjing, China; 5 Journal Publishing Center, Nantong University, Nantong, China; 6 Guangxi University of Chinese Medicine, Nanning, China; 7 Ordos Institute of Technology, Ordos City, China; 8 Xinyang Normal University, Xinyang, China; 9 Yangling Vocational and Technical College, Yangling, China; 10 West Anhui University, Lu'an, China; 11 Faculty of Social Sciences and Liberal Arts, UCSI University, Kuala Lumpur, Malaysia; Goteborg University: Goteborgs Universitet, SWEDEN

## Abstract

**Objective:**

The study aims to examine the impact of physical exercise on sleep quality among college students and elucidate the mediating roles of self-efficacy and emotional control in this relationship.

**Methods:**

Data were obtained from the 2024 China College Students’ Physical Activity and Health Tracking Survey (CPAHLS-CS). A sample of 10,970 college students was included. Physical exercise levels were measured using the Physical Activity Rating Scale-3 (PARS-3), sleep quality was assessed using the Pittsburgh Sleep Quality Index (PSQI), self-efficacy was measured using the General Self-Efficacy Scale (GSES), and emotional control was assessed using the Adolescent Psychological Resilience Scale. Regression analysis and Bootstrap mediation analysis were employed to test the hypothesised relationships.

**Results:**

(1) The direct effect of physical exercise on sleep quality was not significant (β = 0.011, P > 0.05). However, the total effect was negative (β = −0.056, P < 0.001), indicating that indirect effects comprised the predominant mechanism. (2) Self-efficacy (effect size = −0.024) and emotional control (effect size = −0.022) each independently mediated the relationship between physical exercise and sleep quality. (3) In the pathway through which physical exercise influences sleep quality, self-efficacy, and emotional control functioned as independent mediating variables. Specifically, physical exercise indirectly improved sleep quality by enhancing self-efficacy while positively impacting sleep quality through a distinct mediating mechanism involving strengthening emotional control.

**Conclusions:**

This study demonstrates the influence of physical exercise, self-efficacy, and emotional control on sleep quality. The findings suggest that physical exercise indirectly optimises sleep quality through a dynamic and synergistic mechanism involving the enhancement of self-efficacy and emotional control. This study provides theoretical support and practical pathways for sleep quality interventions in college students.

## Introduction

Sleep is essential for maintaining physiological balance and neurocognitive function, with its quality being crucial for overall health [1,2]. Globally, about a quarter of the population reports sleep problems, and approximately 27% suffer from sleep disorders. University students are particularly affected, with sleep quality issues reported in 13.93% to 37.24% of Chinese college students [3]. High sleep quality is positively correlated with life satisfaction [4], academic performance [5,6], quality of life [7], and physical health [8]. In contrast, poor sleep quality increases psychological distress [9], risks of depression and anxiety [10], as well as susceptibility to physical conditions [11] such as hypertension [7], diabetes, and obesity [12]. Existing research has primarily focused on the impact of physical exercise, social relationships, and pharmacological interventions on sleep quality [13–16]. However, social relationships are challenging to standardize, and pharmacological treatments carry dependency risks. Therefore, physical exercise emerges as a more promising and practical intervention strategy [17].

Physical exercise is widely recognized as beneficial for mood, physical health, and overall well-being. In China, 37.2% of residents aged 7 and above engaged in regular physical activity in 2020, an increase of 3.3 percentage points since 2014 [18]. However, college students do not benefit adequately, often facing insufficient exercise, rising obesity, and declining fitness levels [19]. A national survey in 2007 revealed particularly low participation among Chinese youth, with 75.5% of those aged 20–29 exercising less than twice per week [20]. Moderate-to-vigorous physical activity can improve mood regulation [21], immune function [22], sleep quality [23], and psychological resilience, and is negatively correlated with student burnout [24]. Yet, due to low frequency and short duration, college students often fail to gain these benefits. Insufficient physical exercise increases the risk of numerous health issues, including obesity, diabetes, hypertension, certain cancers, depression, coronary artery disease, osteoporosis, and dementia [25].

Self-efficacy, introduced by American psychologist Albert Bandura in 1977, refers to an individual’s belief in their ability to organize and execute the courses of action required to produce given attainments [26]. Individuals with high self-efficacy tend to choose challenging tasks, persist through difficulties, and view stressors as opportunities for growth. They also display stronger motivation, set higher goals, and regulate their emotions and behaviors more effectively [27–30]. In contrast, those with low self-efficacy often avoid challenges, show less persistence, and experience more negative emotions and thought patterns [31,32].In summary, we hypothesize that college students’ self-efficacy and sleep quality are related. Specifically, students with higher self-efficacy are expected to have better sleep quality. Conversely, those with lower self-efficacy are anticipated to experience poorer sleep quality.

Psychological resilience refers to an individual’s ability to effectively cope with, adapt to, and ultimately recover from or surpass their original state when faced with stress, adversity, trauma, or significant life changes [33].Emotional control is one of the five factors of psychological resilience [34]. Individuals with high emotional control effectively manage emotions and find positive meaning in challenges, aiding their recovery [35–37]. Conversely, those with low emotional control struggle to adapt, are more prone to negative emotions like depression, and may experience worsened physical health and treatment outcomes [38,39]. In summary, it is hypothesised that college students with higher emotional control will exhibit better sleep quality, while those with lower emotional control will experience poorer sleep quality.

American psychologist Aaron Beck developed cognitive-behavioural theory (CBT). This theory centres on the interaction of cognition, emotion, and behaviour. CBT’s core tenet is that cognitive processes play a key role in shaping individual feelings and behaviours. [40] Physical exercise improves physical function. This leads to better sleep quality. Better sleep, in turn, encourages a more positive view of one’s health. This positive outlook further strengthens self-efficacy. At the same time, overcoming challenges during exercise changes how people see themselves. It enhances their ability to cope with difficulties. This process boosts both self-efficacy and emotional control. Individuals with high emotional control are better equipped to handle sleep problems and utilize effective coping strategies, leading to further improvements in sleep quality. However, existing studies have largely focused on single mediator variables, lacking in-depth exploration of chain mediation mechanisms. This research, informed by cognitive-behavioural theory (CBT), examines the link between physical exercise and sleep quality. A chain mediation model will be used to understand the roles of self-efficacy and emotional control in this relationship. Ultimately, this study seeks to provide a theoretical foundation for creating effective, multi-faceted interventions.

**Fig 1:**
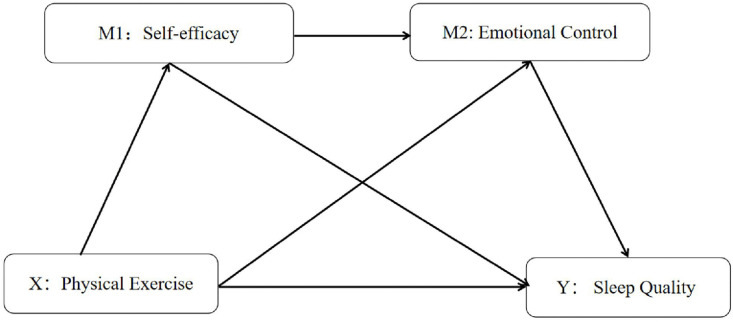
Hypothesized model.

Based on the aforementioned research background, the present study proposes the following hypotheses grounded in CBT. The hypothesized model is depicted in Fig 1 Hypothesized Model.

- ***H1:*** *Physical exercise is positively associated with sleep quality.*
- ***H2:*** *Self-efficacy mediates the relationship between physical exercise and sleep quality.*
- ***H3:*** *Emotional control mediates the relationship between physical exercise and sleep quality.*
- ***H4:*** *Self-efficacy and emotional control sequentially mediate the relationship between physical exercise and sleep quality.*

## Methods

### Participants

This research was a cross-sectional survey.This study aims to explore the complex relationship between physical exercise and sleep quality among university students. To ensure the reliability and representativeness of the results, a stratified, cluster, and staged sampling method was used to select the survey participants. This study utilised data from the 2024 Chinese College Students Physical Activity and Health Longitudinal Survey (CPAHLS-CS). CPAHLS-CS aims to collect a set of high-quality individual-level microdata representing Chinese college students’ physical activity and mental and physical health behaviours. The data is intended to analyse cross-cutting issues related to physical activity and health among Chinese college students and promote interdisciplinary research on college student health problems. CPAHLS-CS has been widely used to analyse the health status of Chinese college students [40–42].This study adheres to the American Psychological Association’s (APA) Ethical Principles of Psychologists and Code of Conduct.The study protocol was approved by the ethics committee at Nantong University and documented under approval number 2022(70). Before commencing the formal investigations and testing, the researchers obtained informed consent from all the participants involved in the study.The survey was conducted using an anonymous completion format. The demographic information collected included school, gender, and academic year. Prior to distributing the questionnaires, the researchers read aloud the introductory section of the survey and informed participants of the relevant consent and procedures. Participants provided verbal consent before receiving the questionnaires. After reviewing the introduction again, those who wished to continue could complete the survey on a fully voluntary basis, and their data would be included in the collection. Participants who were unwilling to take part had the option to withdraw at any time without any penalty, and their information would not be recorded.

The survey participants selected for this study comprised students enrolled in general higher education institutions within mainland China. The recruitment period for the study spanned from 08/10/2024 to 09/11/2024, with the subsequent questionnaire survey being conducted from 11/11/2024 to 24/11/2024. The list of regular universities was based on the Ministry of Education’s “List of National Regular Universities (as of June 20, 2024).”Exclusion criteria were as follows: 1) questionnaires with unrecognisable full school names were removed; 2) questionnaires with at least 21 consecutive identical responses were removed; and 3) questionnaires with completion times in the lowest 0.5% and highest 0.5% were removed, given that the average completion time was 6 minutes and 12 seconds [43]. The study sample was restricted to college students attending higher education institutions located in central mainland China, including Hubei Province, Henan Province, and Jiangxi Province. The survey was administered in November 2024 using the Questionnaire Star platform, distributed uniformly to class groups, and 36,756 questionnaires were ultimately collected. The final sample included 10,970 individuals. Specific demographic information is presented in Sample characteristics table (Table 1).

**Table 1:** Sample characteristics table.

Variable		n	%
Gender			
	Male	4319	39.4
	Female	6651	60.6
Grade			
	Freshman	7810	71.2
	Sophomores	2806	25.6
	Juniors	290	2.6
	Seniors	64	0.6
Total		10970	100.0

### Measures

#### Sociodemographic information.

Sociodemographic information collected in this study included gender, year in school, and age.

#### Physical exercise.

Physical exercise levels were assessed using the Physical Activity Rating Scale-3(PARS-3), developed by Japanese scholar Kimio Hashimoto and revised by Chinese scholars Liang et al. The PARS-3 assesses physical activity volume by considering the intensity, frequency, and duration of exercise and is used to measure physical activity participation53. Each item on the PARS-3 is rated on a 5-point scale, ranging from 1 (never participate in physical exercise) to 5 (frequently participate in physical exercise). Higher scores indicate greater physical activity volume, reflecting the extent of college students’ physical activity participation within a specific timeframe. The raw score from the questionnaire is calculated using the following formula: Physical activity volume score = Intensity × (Duration – 1) × Frequency. Norms for Chinese adults using the PARS-3 are as follows: low exercise volume ≤ 19 points, moderate exercise volume 20–42 points, and high exercise volume ≥ 43 points [44]. The test-retest reliability of the PARS-3 is 0.820, and its applicability to Chinese college student populations has been validated in multiple studies [44–47].

#### Sleep quality.

Sleep quality was assessed using the Pittsburgh Sleep Quality Index (PSQI). The PSQI was developed in 1989 by Dr. Buysse et al., psychiatrists at the University of Pittsburgh. The PSQI is a tool suitable for assessing sleep quality and observing treatment effects in patients with sleep disorders and mental disorders, as well as for conducting sleep quality surveys in the general population and exploring the relationship between sleep quality and psychosomatic health. After Liu’s revision, the scale became more applicable in a Chinese context. The scale contains 23 items divided into 7 subscales, each scored from 0 to 3 points. The cumulative value of the scores of each subscale is the total PSQI score, ranging from 0 to 21 points. Higher total scores indicate poorer sleep quality. The scoring criteria for the total PSQI score are as follows: 0–4 points indicate good sleep quality, 5–7 points indicate fair sleep quality, and 8–21 points indicate poor sleep quality [48]. Scores of 0–4 were categorized as Group 1, scores of 5–7 as Group 2, and scores of 8–21 as Group 3.The scale demonstrates reliability and validity, with a Cronbach’s α coefficient of 0.764 [49].

#### General Self-Efficacy.

The General Self-Efficacy Scale (GSES) was used to measure general self-efficacy among college students [50]. Existing research supports the association between general self-efficacy and changes in physical activity behaviour [51]. The cutoff score for the GSES is 2.5 points; scores below 2.5 points indicate low general self-efficacy, and participants were categorised into “low” and “normal” groups for specific analyses [52]. The Chinese version of the GSES was translated and revised by Wang et al. in 2001, and its reliability and validity were analysed [53]. The results revealed that the GSES has good reliability, with an internal consistency coefficient of Cronbach’s α of 0.87, a test-retest reliability of r = 0.83 (p < 0.001), and a split-half reliability of r = 0.82 (n = 401, p < 0.001).

#### Emotional control.

Psychological resilience was measured using the Adolescent Psychological Resilience Scale, compiled by Hu and Gan, consisting of 27 items across multiple dimensions, including goal orientation, emotional control, positive cognition, family support, and interpersonal assistance. Each item adopts the 5-point Likert scale, from “completely disagree” to “completely agree,” scoring 1–5 points respectively. The average score of all items was calculated, with higher scores indicating greater emotional control. In this study, the Cronbach’s α coefficient for the scale was 0.84 [34].This study used the emotional control subscale for measurement. It serves as a core active mechanism of psychological resilience during stress coping. It also functions as a key internal resource for regulating emotional responses in adversity. Thus, it effectively represents the central component of overall resilience [54,55].

### Statistical analysis

Statistical analysis was performed using SPSS 26.0 and Excel software. The steps were as follows: 1) Data obtained from Questionnaire Star were pre-processed using Excel software, and missing or problematic data were retested or deleted. 2) Harman’s single-factor test was used to examine common method bias to avoid common method variance issues. 3) Chi-square tests were used to analyse differences in physical activity between students of different genders and years. Cramer’s V coefficient was used to reveal the strength of association between variables. Cramer’s V ranges from 0 to 1, with higher values indicating stronger correlations. A Cramer’s V coefficient > 0.1 indicates a weak correlation between categorical variables; a Cramer’s V coefficient > 0.3 indicates a moderate correlation; and a Cramer’s V coefficient > 0.5 indicates a high correlation [56]. ANOVA was used to analyse physical exercise, sleep quality, general self-efficacy, and emotional control The η² value ranges from 0 to 1; according to Cohen’s criteria, 0.01 represents a small effect, 0.06 represents a medium effect, and 0.14 represents a large effect [57]. 4) Pearson correlation analysis was used to examine the correlations between physical exercise, sleep quality, self-efficacy, and emotional control among college students. 5) Mediation analysis was performed using regression analysis, and PROCESS macro was used for multiple regression analysis, with bootstrapping to test the mediation effects.

Because subjective scales were used to collect data in this study, Harman’s single-factor test was conducted to examine common method bias. Exploratory factor analysis was performed on all questionnaire items for physical exercise, self-efficacy, emotional control, and sleep quality. The results showed that three principal components were extracted with eigenvalues greater than 1, and the largest factor explained 39.9% of the variance, below the commonly set threshold of 40%. Therefore, common method bias was not a concern in this study.

## Results

### Descriptive analysis

The study included 10,970 participants, of whom 4,319 were male (39.4%) and 6,651 were female (60.6%). As shown in Table 2, there were 7,810 freshmen (71.3%), 2,806 sophomores (25.6%), and smaller sample sizes for juniors and seniors, with 290 (2.6%) and 64 (0.6%), respectively. Low exercise volume accounted for 71.3% (n = 7,819), moderate exercise volume for 16.6% (n = 1,822), and high exercise volume for 12.1% (n = 1,329). Chi-square tests revealed a strong association between gender and exercise volume (χ² = 14660.06, p < 0.001, Cramer’s V = 0.366). The proportion of males with high exercise volume was significantly higher than that of females, while the proportion of females with low exercise volume reached 84.0%, higher than 51.6% for males. Exercise volume showed polarisation with increasing year in school: the proportion of seniors with low exercise volume sharply decreased to 37.5%, while the proportion with high exercise volume surged to 43.8% (χ² = 247.248, p < 0.001, V = 0.106), possibly related to changes in academic pressure. For sleep quality, “very good” accounted for 25.6% (n = 2,808), “good” for 49.5% (n = 5,433), “fair” for 21.1% (n = 2,314), and “poor” for 3.8% (n = 415). The proportion of males with “poor” sleep quality was significantly higher than that of females, but the effect size was small (χ² = 25.372, p < 0.001, V = 0.048). The proportion of sophomores with “fair/poor” sleep quality reached 28.8%, higher than the 23.1% for first-year students. The proportion of seniors with “poor” sleep quality reached 6.3% (χ² = 97.386, p < 0.001, V = 0.054), possibly related to graduation stress. For self-efficacy, low self-efficacy accounted for 90.3% (n = 9,904), while normal self-efficacy accounted for only 9.7% (n = 1,066). The proportion of males with high self-efficacy was significantly higher than that of females (χ² = 90.645, p < 0.001, V = 0.091). High self-efficacy showed an increasing trend with increasing years in school: 13.4% for juniors and 15.6% for seniors, but the year difference only reached marginal significance (χ² = 7.525, p = 0.057, V = 0.026), requiring validation with a larger sample of upper-level students.

**Table 2:** Descriptive statistics and Chi-square tests for categorical variables.

Characteristic			number	Exercise Volume				Sleep Quality					Self-Efficacy		
				Low	Moderate	High	*Statistics*	Very Good	Good	Fair	Poor	*Statistics*	Low	Normal	*Statistics*
**Overall**		n	10970	7819	1822	1329		2808	5433	2314	415		9904	1066	
		%	100	71.3	16.6	12.1		25.6	49.5	21.1	3.8		90.3	9.7	
**Gender**															
	Male	n	4319	2229	1060	1030	X^2^ = 14660.060 ***P*** < 0.001 V = 0.366	1046	2126	941	206	X^2^ = 25.372 ***P*** < 0.001 V = 0.048	3755	564	X^2^ = 90.645 ***P*** < 0.001 V = 0.091
		%	100	51.6	24.5	23.8		24.2	49.2	21.8	4.8		86.9	13.1	
	Female	n	6651	5590	762	299		1762	3307	1373	209		6149	502	
		%	100	84.0	11.5	4.5		26.5	49.7	20.6	3.1		92.5	7.5	
Grade															
	Freshman	n	7810	5566	1341	903	X^2^ = 247.248 ***P*** < 0.001 V = 0.106	2169	3843	1535	263	X^2^ = 97.386 ***P*** < 0.001 V = 0.054	7067	743	X^2^ = 7.525 ***P*** = 0.057 V = 0.026
		%	100	71.3.	17.2	11.6		27.8	49.2	19.7	3.4		90.5	9.5	
	Sophomore	n	2806	2098	414	294		569	1429	676	132		2532	274	
		%	100	74.8	14.8	10.5		20.3	50.9	24.1	4.7		90.2	9.8	
	Junior	n	290	131	55	104		54	135	85	16		251	39	
		%	100	45.2	19.0	35.9		18.6	46.6	29.3	5.5		86.6	13.4	
	Senior	n	64	24	12	28		16	26	18	4		54	10	
		%	100	37.5	18.8	43.8		25.0	40.6	28.1	6.3		84.4	15.6	

As shown in Table 3, the overall mean emotional control score for college students was 19.087 ± 4.552, with significant differences observed at both the gender and year levels (p < 0.001). Regarding gender, male students had a significantly higher mean emotional control score (20.726 ± 4.649) than female students (19.211 ± 4.387). Regarding year in school, senior students had a significantly higher mean emotional control score (21.438 ± 4.846) compared to junior (20.186 ± 4.965), freshman (19.911 ± 4.596), and sophomore (19.442 ± 4.531) students.

**Table 3:** Overview of descriptive analysis results.

Characteristic		Statistic	Emotional Control
		M	19.087
		SD	4.552
	Male	M	20.726
Gender		SD	4.649
	Female	M	19.211
		SD	4.387
	η^2^		0.026
	F		297.866
	***P***		<0.001
	Freshman	M	19.911
		SD	4.596
	Sophomores	M	19.442
Grade		SD	4.351
	Juniors	M	20.186
		SD	4.965
	Seniors	M	21.438
		SD	4.846
	η^2^		0.003
	F		10.819
	***P***		<0.001

### Correlation analysis

As shown in Table 4, there was a significant negative correlation between physical exercise and sleep quality (r = −0.027). A significant positive correlation existed between physical exercise and self-efficacy (r = 0.223). A significant positive correlation was found between physical exercise and emotional control (r = 0.212). Sleep quality and self-efficacy had a significant negative correlation (r = −0.196). Sleep quality and emotional control had a significant negative correlation (r = −0.269). A significant positive correlation existed between self-efficacy and emotional control (r = 0.416).

**Table 4:** Correlation analysis.

Variable	X	Y	M1	M2
X_Physical Exercise	1			
Y_Sleep Quality	−0.027**	1		
M1_Self-Efficacy	0.223**	−0.196**	1	
M2_Emotional Control	0.212**	−0.269**	0.416**	1

*Note*. ** indicates significance at *p* < 0.01 (two-tailed).

### Regression analysis

To further test the hypothesised chain mediation effects of self-efficacy and emotional control in the relationship between physical exercise and sleep quality, a chain mediation analysis was conducted using Model 6 in the PROCESS macro for SPSS 27.0, with gender and year in school as covariates. The results, presented in Table 5, indicate that after controlling for gender and year, physical exercise positively predicted self-efficacy (β = 0.222, *p* < 0.001) and positively predicted emotional control (β = 0.093, *p* < 0.001). Self-efficacy positively predicted emotional control (β = 0.388, *p* < 0.001) and negatively predicted sleep quality (β = −0.110, *p* < 0.001). emotional control negatively predicted sleep quality (β = −0.238, *p* < 0.001).

**Table 5:** Regression analysis of the relationships among variables in the Model.

Regression Equation		Overall Model Fit			Regression Coefficient Significance		
Outcome Variable	Predictor Variable	*R*	*R* ^*2*^	*F*	*β*	*SE*	*t*
Sleep Quality		0.311	0.097	234.076***			
	Physical Exercise				0.011	0.010	1.092
	Self-Efficacy				−0.110	0.010	−10.859***
	Emotional Control				−0.238	0.010	−23.496***
	Gender				−0.175	0.020	−8.721***
	grade				0.156	0.016	9.489***
Self-Efficacy		0.224	0.050	192.754***			
	Physical Exercise				0.222	0.010	22.108***
	Gender				0.000	0.021	−0.001
	grade				0.034	0.017	2.001
Emotional Control		0.445	0.198	675.322***			
	Physical Exercise				0.093	0.009	9.883***
	Self-Efficacy				0.388	0.008	44.249***
	Gender				−0.199	0.019	−10.554***
	Grade				−0.067	0.016	−4.343***

Note: ***P < 0.001.

### Mediation analysis

As shown in Table 6, the 95% confidence interval (CI) for the indirect effect of physical exercise on sleep quality through self-efficacy was [−0.030, −0.019], which does not include zero. Therefore, self-efficacy significantly mediated the relationship between physical exercise and sleep quality in this study. The 95% CI for the indirect effect of physical exercise on sleep quality through emotional control was [−0.027, −0.017], which also does not include zero, indicating that emotional control significantly mediated the relationship between physical exercise and sleep quality. Furthermore, the 95% CI for the chain mediation effect of physical exercise on sleep quality through self-efficacy and emotional control was [−0.024, −0.018], indicating a significant chain mediation effect.

**Table 6:** Analysis of the mediating effects.

Effect	Value	*BootSE*	*LLCI*	*ULCI*
Total Effect	−0.056	0.010	−0.076	−0.036
Direct Effect	0.011	0.010	−0.009	0.031
Indirect Effect	−0.07	0.004	−0.075	−0.059
Physical Exercise → Self-Efficacy → Sleep Quality	−0.024	0.003	−0.030	−0.019
Physical Exercise → Emotional Control → Sleep Quality	−0.022	0.003	−0.027	−0.017
Physical Exercise → Self-Efficacy → Emotional Control → Sleep Quality	−0.021	0.002	−0.024	−0.018

**Fig 2:**
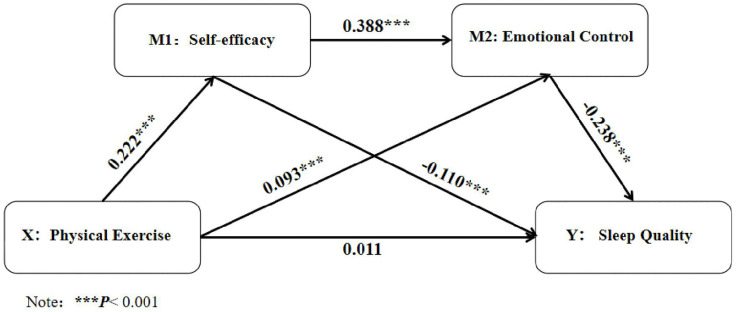
Path analysis diagram.

As shown in Fig 2 Path analysis diagram, the path coefficient from physical exercise to self-efficacy was 0.222, *p* < 0.001, indicating a significant positive effect of physical exercise on self-efficacy. The path coefficient from physical exercise to emotional control was 0.093, *p* < 0.001, indicating a significant positive impact of physical exercise on emotional control. The path coefficient from physical exercise to sleep quality was 0.011, *p* = 0.275, indicating a small and non-significant direct impact of physical exercise on sleep quality. The path coefficient from self-efficacy to emotional control was 0.388, *p* < 0.001, indicating a significant positive effect of self-efficacy on emotional control. The path coefficient from self-efficacy to sleep quality was −0.110, *p* < 0.001, indicating a significant negative impact of self-efficacy on sleep quality. The path coefficient from emotional control to sleep quality was −0.238, *p* < 0.001, indicating a significant negative effect of emotional control on sleep quality.

## Discussion

Based on social cognitive theory, this study aimed to elucidate how physical exercise influences sleep quality in college students by introducing self-efficacy and emotional control as mediating variables. The results showed that the total effect of physical exercise on sleep quality was primarily mediated through the serial pathways involving self-efficacy and emotional control. In contrast, the direct effect was not statistically significant. These results indicate that physical exercise can trigger a series of positive changes. First, physical exercise can increase self-efficacy. Second, this increased self-efficacy encourages the development of emotional control. Finally, this enhanced resilience, thanks to its effect on emotional regulation, results in optimized sleep quality. The validation of this serial pathway has dual theoretical value. First, it expands the application of social cognitive theory in health behaviour, confirming that behavioural interventions can indirectly improve health outcomes through the dynamic interaction of cognitive restructuring and emotional regulation. Furthermore, this study highlights the importance of emotional control. The mediation model is crucial in linking cognitive resources to adaptive behaviour. This provides a valuable analytical framework for understanding how physical exercise affects sleep quality. This research offers valuable insights for practical applications. It provides evidence-based recommendations for interventions to improve sleep quality among college students.

### Descriptive results analysis

Descriptive results from various variables indicate significant differences in sleep quality among college students across gender and grade levels. In terms of gender, female students exhibited higher sleep quality than male students, which contradicts previous research findings. College students are in a transitional phase from familial supervision to self-management, and their behavioral patterns are characterized by distinct age-related features. Male students may more frequently engage in nighttime activities such as video gaming, social media use, and social interactions, which can directly lead to delayed sleep onset, irregular sleep schedules, and consequently, reduced sleep quality [58]. In contrast, female students’nighttime activities tend to be more subdued, which may facilitate sleep initiation. In terms of grade level, lower-grade students demonstrate better sleep quality than their upper-grade counterparts, a finding consistent with previous studies [59]. This disparity is mainly driven by escalating academic and career pressures. These pressures intensify with each year. Upper-grade students typically face multiple stressors, including preparing for graduate school entrance exams, job hunting, and completing graduation projects.The persistent psychological stress associated with these demands can easily trigger anxiety, which directly leads to difficulties in falling asleep and maintaining sleep [60].Furthermore, physical exercise levels also varied significantly by gender and grade among college students. In terms of gender, male students reported higher physical exercise levels than females. This finding is consistent with existing research [61]. Traditional gender roles often link sports with masculine traits like strength and vitality. This association encourages greater male participation. Conversely, female students may internalize societal expectations of femininity, which emphasize grace and reserve. Concerns about sweating and body exposure during exercise may also reduce their participation willingness and frequency [62]. At the grade level, upper-year students demonstrate higher physical activity levels than their lower-year counterparts. This finding contradicts previous research [63]. The observed pattern may be explained by considering the developmental challenges faced by different student groups. Lower-year students experience dual pressures of academic adaptation and social restructuring. Their limited self-efficacy in time management creates difficulties in systematically maintaining regular exercise [64]. As students advance through their academic journey, they accumulate psychological resources through successfully navigating various challenges. These accumulated resources enable better recognition of physical exercise’s value in stress buffering and emotion regulation [65]. This enhanced understanding leads students to proactively incorporate exercise into their daily routines.

However, the current study found a non-significant direct effect of physical exercise on sleep quality, and Hypothesis H1 was not supported. This contradicts previous research findings [66]. This discrepancy may be attributed to inappropriate dietary habits or poor sleep environments that counteract the potential physiological benefits of exercise [67]. Previous studies have shown that eating within two hours before bedtime, especially high-calorie foods, increases the risk of obesity. Obese individuals are more likely to develop gastroesophageal reflux disease (GERD), which can lead to nocturnal awakenings, chest discomfort, and reduced sleep quality. Additionally, caffeine consumption can also negatively impact sleep [68]. Research has shown that caffeine intake can cause temporary alertness, reduced fatigue, prolonged sleep latency, shortened sleep duration, reduced sleep efficiency, and poorer perceived sleep quality [67]. Furthermore, the quality of the sleep environment plays a role in sleep quality. Studies have shown that inadequate sleep environments are associated with lower sleep quality, while better sleep environments are associated with higher sleep quality [69].

### Negative mediating effect of self-efficacy

It’s important to note that the direct effect of physical exercise on sleep quality was not statistically significant in this study. While seemingly contradictory, this result indicates that other, more complex mechanisms may be at play. Specifically, cognitive pathways might mediate physical exercise interventions’ health benefits. The study focused on analysing the mediating mechanisms to address this theoretical puzzle. The results showed that self-efficacy had a significant negative mediating effect on the relationship between physical exercise and sleep quality, supporting Hypothesis H2. Self-efficacy showed significant correlations. It was positively correlated with physical exercise. It was also negatively correlated with sleep quality. This means self-efficacy mediates how physical exercise influences sleep quality. We examined self-efficacy as a mediating factor to better understand the link between physical exercise and sleep quality. Results showed that self-efficacy’s mediating effect actually boosted the positive impact of exercise on sleep. This finding is in line with existing research. Individuals with healthy self-efficacy can more effectively manage fatigue, pain, and emotional distress, reducing the interference of psychological stress in sleep [70].

Physical exercise can indirectly improve sleep quality by enhancing self-efficacy. Regular physical exercise, by achieving stage goals, enhances an individual’s confidence in their abilities [71]. This confidence can be transferred to stress management, helping college students cope more calmly with academic challenges and reducing the interference of stress hormones on the sleep cycle. Individuals with healthy self-efficacy have stronger behavioural control. They will proactively develop sleep schedules and reduce phone use before bed, avoiding blue light inhibition of melatonin secretion, which can lead to delayed sleep onset [72]. When faced with academic stress, they are more likely to adopt positive coping strategies such as exercise-related stress reduction, reducing the interference of nighttime anxiety on sleep [41]. The factors involved create a synergistic effect. This strengthens how self-efficacy acts as a mediator between physical exercise and sleep quality. This leads to a positive cycle, ultimately enhancing sleep. Improved sleep is crucial for individuals’ overall health and well-being, especially students, promoting physical and mental health.

### Negative mediating effect of psychological resilience

The enhancement of self-efficacy is only the primary stage of cognitive transformation. emotional control, as the core ability of emotional regulation, may play a secondary transformation role in this process. Data analysis showed that physical exercise significantly affected sleep quality through the negative mediating pathway of emotional control, supporting Hypothesis H3. This is consistent with previous research [73]. A significant finding was the higher path coefficient for this specific pathway. This was compared to the self-efficacy pathway. This difference implies that the psychological benefits of physical exercise interventions evolve over time. The progression is from improving cognitive function to enhancing emotional adaptation. Previous studies have shown that emotional control is beneficial. Resilient individuals use active and effective coping strategies when facing stress. They avoid overthinking negative outcomes. They also reduce nighttime ruminative thinking. This helps them shorten sleep onset time and improve sleep quality [74].

Participating in moderate physical exercise can effectively improve the emotional control of college students. Physically, exercise promotes the secretion of neurotransmitters such as dopamine [75]. These substances improve mood and enhance an individual’s ability to cope with stress, thereby improving emotional control. College students can benefit from aerobic exercises like dancing and running. These activities provide a sense of pleasure and achievement. This experience boosts self-confidence. It also builds emotional control, making them better able to handle difficulties. When faced with academic stress and interpersonal relationship problems, college students who regularly participate in physical exercise may show a stronger ability to adapt psychologically and can recover more quickly from setbacks [76]. emotional control improves sleep quality by constructing an emotion-coping synergy system. In terms of emotion regulation, psychologically resilient individuals are better at inhibiting anxiety. This prevents negative emotions from affecting their sleep, ensuring that they don’t carry these negative feelings into the sleep period. At the same time, on the coping strategy side, the proportion of problem-oriented strategies used by these individuals is significantly higher than that of emotion-oriented strategy, and this strategic choice advantage enables them to resolve daytime stressors, reducing the duration of nighttime ruminative thinking. The synergy of these two factors ultimately optimises sleep quality by reducing pre-sleep physiological arousal levels [74].

### Serial mediation effect of self-efficacy and emotional control

This study found that the direct effect of physical exercise on college students’ sleep quality was insignificant. Still, physical exercise indirectly improved sleep quality through the serial mediation pathway of self-efficacy and emotional control, supporting Hypothesis H4. The mediating effects in this study were relatively small. This may be due to the long causal chain from physical exercise to sleep quality. It may also involve complex psychological mechanisms. Still, this finding has important theoretical value. It clearly shows how physical exercise improves sleep. This happens through a chain of factors: first self-efficacy, then emotional control. The finding also has practical significance. Even with a small effect, encouraging exercise can be a low-cost strategy. It can improve both psychological health and sleep quality. At the population level, these benefits could add up to meaningful public health impacts. This result challenges traditional research’s linear assumption that physical exercise directly promotes sleep quality [66]. Correlation analysis showed that self-efficacy and emotional control were significantly correlated with physical exercise and sleep quality, verifying the accuracy of the serial mediation model. Physical exercise positively influences self-efficacy, enhancing emotional control and promoting sleep quality in college students. A comprehensive analysis of the above mediation pathways shows that improving sleep quality through physical exercise is a synergistic upgrade of the cognitive and emotional systems.

According to Bandura’s social cognitive theory, self-efficacy is the core driving force for behaviour change. Regular physical exercise has a positive effect on enhancing an individual’s self-efficacy. Individuals constantly challenge their physical limits and break through exercise goals regularly, improving their confidence in physical control and life management [71]. Achievement experiences such as completing a long run or learning complex fitness exercises can strengthen self-efficacy. Healthy self-efficacy prompts individuals to adopt a proactive approach to challenges, treating challenges as opportunities for growth and continuously enhancing emotional control [50]. Individuals with healthy self-efficacy respond actively when faced with academic stress and interpersonal problems. They seek solutions to their problems. They also exercise their psychological adaptation abilities. This leads to a continuous enhancement of their emotional control. The enhancement of emotional control has a significant positive effect on sleep quality. Psychologically, individuals with strong emotional control can better manage stress and negative emotions [77]. When dealing with stress, these individuals avoid excessive worrying and are adept at managing their emotions. This proactive approach prevents negative emotions from interfering with sleep at night. It also lowers the risk of developing sleep disorders like insomnia. Consequently, they enjoy good sleep quality. Therefore, physical exercise improves sleep quality by enhancing self-efficacy and emotional control. This chain of action provides an important basis for understanding the impact of physical exercise on individual physical and mental health. Also, it provides a feasible way to improve sleep quality and overall health.

### Limitations

This study has limitations: 1) The tests only used retrospective scales and did not include instrumental measurements. 2) Regarding variable selection, this study did not comprehensively cover the factors affecting college students’ physical exercise and sleep quality. 3) Causal inference is limited by the cross-sectional nature of this study. Future research should consider methodological improvements. This includes expanding the range of variables. It also involves introducing new measurement techniques or tools. Moreover, research should account for external environmental influences and individual characteristics. A comprehensive analysis should integrate these findings with existing knowledge.

## Conclusion

The direct effect of physical exercise on sleep quality was not statistically significant. Self-efficacy and emotional control independently mediated the relationship between physical exercise and sleep quality. The chain mediation effect was substantial. Physical exercise improves sleep quality by enhancing emotional control through promoting self-efficacy.

## Abbreviations

CBT: Cognitive-Behavioral Theory
CPAHLS-CS: Chinese College Students’ Physical Activity and Health Tracking Survey
GSES: General Self-Efficacy Scale
PARS-3: Physical Activity Rating Scale-3
PSQI: Pittsburgh Sleep Quality Index
